# Severe Respiratory Illness Associated with Human Metapneumovirus in Nursing Home, New Mexico, USA

**DOI:** 10.3201/eid2502.181298

**Published:** 2019-02

**Authors:** Sandra A. Peña, Sarah Shrum Davis, Xiaoyan Lu, Senthil Kumar K. Sakthivel, Teresa C.T. Peret, Erica Billig Rose, Chad Smelser, Eileen Schneider, Nimalie D. Stone, John Watson

**Affiliations:** New Mexico Department of Health, Santa Fe, New Mexico, USA (S.A. Peña, S. Shrum Davis, C. Smelser);; Centers for Disease Control and Prevention, Atlanta, Georgia, USA (X. Lu, S.K.K. Sakthivel, T.C.T. Peret, E. Billig Rose, E. Schneider, N.D. Stone, J. Watson)

**Keywords:** human metapneumovirus, HMPV, viruses, outbreak, severe respiratory illness, respiratory infections, nursing home, infection control, New Mexico, United States

## Abstract

Human metapneumovirus is an emerging pathogen that causes upper and lower respiratory illness. Nursing home outbreaks of infection with this virus can cause severe illness and lead to poor patient outcomes. We report an outbreak investigation in a nursing home during 2018 and infection control guidelines to assist in disease control.

Human metapneumovirus (HMPV) is an enveloped, single-stranded RNA virus first described in 2001. Almost all children are infected by 5 years of age, but reinfection can occur throughout life, especially in older adults and immunocompromised persons (*1**–**6*). In a study of prospective adult cohorts covering 4 consecutive winters, annual HMPV detection varied (3%–3.3% in the first and third years and 6%–7.1% in the second and fourth years) (*5*). In the United States, HMPV circulates seasonally from winter through late spring (*7*). There is no vaccine; treatment is supportive.

On February 22, 2018, a nursing home in New Mexico contacted the New Mexico Department of Health to report acute respiratory illness among 5 residents who had negative rapid diagnostic influenza test results. This rural, 86-bed facility (44 resident rooms [2 residents/room] in 5 contiguous areas, 2 dining rooms, 2 activity rooms, and 120 staff) provides long-term care, short-term rehabilitation, skilled nursing services, and dementia care. The New Mexico Department of Health initiated an investigation to determine the cause of the illness, characterize the clinical manifestations, and limit transmission.

A case of respiratory illness was defined as illness in a nursing home resident with onset during February 15–March 31, 2018, and comprising >1 of the following signs and symptoms: cough, fever, shortness of breath, or hypoxia (oxygen saturation <90%). A total of 49 (62%) of 79 residents were identified as case-patients (Figure); 17 (35%) were men, and the median age was 81 years (range 55–99 years). Signs and symptoms reported were cough (73%), temperature >99.1°F (72%), hypoxia (33%), shortness of breath (27%), sore throat (8%), nasal congestion (8%), myalgia (6%), headache (6%), and wheezing (4%).

**Figure:**
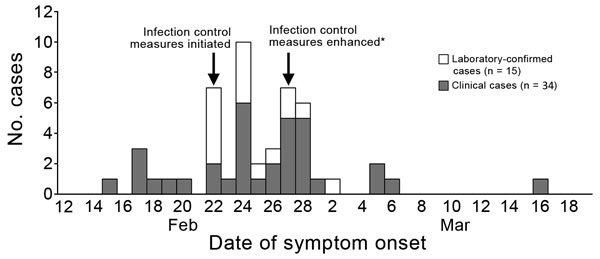
Distribution of 49 cases of a human metapneumonvirus–associated severe respiratory illness in a nursing home, New Mexico, USA, February–March 2018. *Cohorting of cases enhanced.

The most common underlying medical conditions among the 49 case-patients were heart disease (69%), dementia (63%), other neurologic disease (25%), diabetes (18%), chronic lung disease (14%), and cancer (10%). Eighteen case-patients (37%) were in hospice or receiving palliative care. Pneumonia was clinically diagnosed in 9 (18%) case-patients and confirmed by chest radiograph. Three case-patients (6%) visited an emergency department and 9 (18%) were hospitalized (median length of stay 7.3 days); none were intubated or admitted to the intensive care unit.

We collected nasopharyngeal or nasopharyngeal/oropharyngeal swab specimens from 38 (78%) of the 49 case-patients within a median of 3 days of symptom onset. Testing at a commercial laboratory detected HMPV in 5 (71%) of 7 nasopharyngeal specimens by using the BioFire Respiratory Panel (20 targets), a reverse transcription PCR (RT-PCR) kit (BioFire Diagnostics, https://www.biofiredx.com). No other pathogens were detected. Thirty-one additional nasopharyngeal/oropharyngeal specimens were sent to the Centers for Disease Control and Prevention (Atlanta, GA, USA) and tested by using a singleplex real-time RT-PCR for HMPV (*8*). HMPV was detected in 10 (32%) of 31 specimens. All 10 HMPV-positive specimens tested were characterized and found to be HMPV genotype A, subtype 2 (*9*). Laboratory confirmation of HMPV was obtained for 15 (39%) of 38 case-patients tested. Respiratory infection was considered contributory to the deaths of 4 (8%) case-patients; 3 were tested by RT-PCR, and 2 were HMPV positive.

In addition to standard precautions, other infection control interventions were implemented at the nursing home, including instituting contact and droplet precautions with either use of single rooms or cohorting cases for 10 days (≈2 incubation periods); suspending new admissions; restricting visitation; canceling group activities, including serving meals in the dining hall; intensifying hand hygiene and environmental cleaning; and extensive education for facility staff. Among staff, 8 (<7%) of 120 self-reported respiratory symptoms during the outbreak. Staff who reported respiratory symptoms were furloughed. No laboratory testing was performed for symptomatic staff. Staff illnesses resulted in an average of 3 days of work missed per ill employee. Challenges identified during the outbreak response included maintaining adequate staffing, maintaining supplies necessary for implementation of infection control precautions, the psychological hardship of restricting residents to their rooms, and difficulties controlling the movement of residents with dementia. The last reported case was on March 16, 2018.

Prompt identification of the pathogen within 2–3 days of specimen collection assisted providers and the facility in coordinating an effective response. Anecdotally, facility staff noted that pathogen identification also promoted credibility. Although diagnostic laboratory testing can be an additional expense, pathogen identification can be invaluable and cost can be reduced by not testing every case-patient once a pathogen has confidently been identified and other pathogens have been ruled out.

We report a large outbreak of HMPV-associated severe respiratory illness in a nursing home that affected 62% of residents. Respiratory illness outbreaks in nursing homes present unique challenges because of needs of the residents and structural configuration of the facility, which must be considered when implementing infection control measures. This outbreak demonstrates the need for considering and testing for HMPV during respiratory outbreaks in nursing homes and other residential care settings. Prompt recognition of respiratory outbreaks and institution of outbreak control measures are key to preventing disease spread, hospitalizations, and deaths.

## References

[1] Haas LE, Thijsen SF, van Elden L, Heemstra KA. Human metapneumovirus in adults. Viruses. 2013;5:87–110. 10.3390/v5010087 23299785 PMC3564111

[2] Widmer K, Zhu Y, Williams JV, Griffin MR, Edwards KM, Talbot HK. Rates of hospitalizations for respiratory syncytial virus, human metapneumovirus, and influenza virus in older adults. J Infect Dis. 2012;206:56–62. 10.1093/infdis/jis309 22529314 PMC3415933

[3] Hamelin ME, Côté S, Laforge J, Lampron N, Bourbeau J, Weiss K, et al. Human metapneumovirus infection in adults with community-acquired pneumonia and exacerbation of chronic obstructive pulmonary disease. Clin Infect Dis. 2005;41:498–502. 10.1086/431981 16028158

[4] Boivin G, DeSerres G, Hamelin ME, Cote S, Argouin M, Tremblay G, et al. An outbreak of severe respiratory tract infection due to human metapneumovirus in a nursing home. Clin Infect Dis. 2007;44:1152–8. 10.1086/513204 17407031

[5] Walsh EE, Peterson DR, Falsey AR. Another piece of the puzzle: human metapneumovirus infections in adults. Arch Intern Med. 2008;168:2489–96. 10.1001/archinte.168.22.2489 19064834 PMC2783624

[6] Liao RS, Appelgate DM, Pelz RK. An outbreak of severe respiratory tract infection due to human metapneumovirus in a long-term care facility for the elderly in Oregon. J Clin Virol. 2012;53:171–3. 10.1016/j.jcv.2011.10.010 22078146

[7] Centers for Disease Control and Prevention. The National Respiratory and Enteric Virus Surveillance System (NREVSS)—human metapneumovirus national trends. Atlanta: the Centers [cited 2018 Aug 15]. https://www.cdc.gov/surveillance/nrevss/hmpv/natl-trend.html.

[8] Sakthivel SK, Whitaker B, Lu X, Oliveira DB, Stockman LJ, Kamili S, et al. Comparison of fast-track diagnostics respiratory pathogens multiplex real-time RT-PCR assay with in-house singleplex assays for comprehensive detection of human respiratory viruses. J Virol Methods. 2012;185:259–66. 10.1016/j.jviromet.2012.07.010 22796035 PMC7119496

[9] van den Hoogen BG, Herfst S, Sprong L, Cane PA, Forleo-Neto E, de Swart RL, et al. Antigenic and genetic variability of human metapneumoviruses. Emerg Infect Dis. 2004;10:658–66. 10.3201/eid1004.030393 15200856 PMC3323073

